# Bladder cancer-derived exosomal KRT6B promotes invasion and metastasis by inducing EMT and regulating the immune microenvironment

**DOI:** 10.1186/s12967-022-03508-2

**Published:** 2022-07-06

**Authors:** Qiang Song, Hao Yu, Yidong Cheng, Jie Han, Kai Li, Juntao Zhuang, Qiang Lv, Xiao Yang, Haiwei Yang

**Affiliations:** Department of Urology, The First Affiliated Hospital of Nanjing Medical University, Nanjing, 210029 People’s Republic of China

**Keywords:** Bladder cancer, Exosome, krt6b, Epithelial–mesenchymal transition, Immune response, Prognosis marker

## Abstract

**Background:**

Tumour-derived exosomes have recently been shown to participate in the formation and progression of different cancer processes, including tumour microenvironment remodelling, angiogenesis, invasion, metastasis, and drug resistance. However, the function and mechanism of exosome-encapsulated nucleic acids and proteins in bladder cancer remain unclear. This study aimed to investigate the effects of tumour-derived exosomes on the tumorigenesis and development of bladder cancer.

**Methods:**

In this study, gene expression profiles and clinical information were collected from The Cancer Genome Atlas (TCGA) database and two independent Gene Expression Omnibus (GEO) datasets. The nucleic acids and proteins encapsulated in bladder cancer-derived exosomes were obtained from the ExoCarta database. Based on these databases, the expression patterns of exosomal mRNAs and proteins and the matched clinicopathological characteristics were analysed. Furthermore, we carried out a series of experiments to verify the relevant findings.

**Results:**

A total of 7280 differentially expressed mRNAs were found in TCGA data, of which 52 mRNAs were shown to be encapsulated in bladder cancer-derived exosomes. Survival analysis based on the UALCAN database showed that among the top 10 upregulated and the top 10 downregulated exosomal genes, only the expression of KRT6B had a statistically significant effect on the survival of bladder cancer patients. Additionally, clinical correlation analysis showed that the elevated level of KRT6B was highly associated with bladder cancer stage, grade, and metastasis status. GSEA revealed that KRT6B was involved not only in epithelial–mesenchymal transition-related pathways but also in the immune response in bladder cancer. Ultimately, our experimental results were also consistent with the bioinformatic analysis.

**Conclusion:**

KRT6B, which can be detected in bladder cancer-derived exosomes, plays an important role in the epithelial–mesenchymal transition and immune responses in bladder cancer. Further research will enable its potentially prognostic marker and therapeutic target for bladder cancer.

**Supplementary Information:**

The online version contains supplementary material available at 10.1186/s12967-022-03508-2.

## Introduction

As one of the most frequently diagnosed cancers worldwide, bladder cancer (BLCA) accounts for nearly 170,000 deaths worldwide annually [1]. Environmental or occupational exposure to carcinogens, especially tobacco, is reported to be the main risk factor for BLCA. BLCA is generally characterized as a heterogeneous disease of two major subtypes, nonmuscle-invasive bladder cancer (NMIBC) and muscle-invasive bladder cancer (MIBC), depending on whether it infiltrates the bladder muscle layer [2]. The two subtypes have unique pathological features and different molecular characteristics. As the most common malignant tumour in the urinary system, there is still no effective method for the diagnosis of BLCA. Cystoscopy biopsy is known as the “gold standard” for the diagnosis of BLCA, but it is an invasive examination [3]. Therefore, it is urgent to find other effective biomarkers to assist in the diagnosis of BLCA.

Exosomes are 30–150 nm extracellular vehicles (EVs) with a variety of biological functions that exist in various biological fluids [4]. Previous studies have shown that exosomes participate in intercellular communication and influence the surrounding microenvironment in numerous tumours, depending on the proteins and nucleic acids they carry [5]. Additionally, tumour-derived exosomes (TDEs) appear to be important regulatory factors in many processes of tumours, including tumour invasion, metastasis, and drug resistance [6]. However, little is known about their roles in BLCA [7]. Therefore, we believe it is necessary to further explore the mechanism of TDEs involved in the tumorigenesis and development of BLCA.

In our study, we collected mRNAs and proteins of BLCA-derived exosomes and analysed the sequencing data of BLCA from The Cancer Genome Atlas (TCGA) database and two independent Gene Expression Omnibus (GEO) datasets. We found that KRT6B was significantly overexpressed in tumour tissues of BLCA patients compared with healthy controls. Additionally, the results of the clinical correlation analysis showed that the expression of KRT6B was closely related to clinical stage, tumour invasion, and metastasis in BLCA patients. By further exploring the possible molecular mechanism of KRT6B in BLCA, we found that it was involved in the regulation of epithelial–mesenchymal transition (EMT) and immune-related pathways. Recently, increasing attention has been given to the role of macrophages in tumour immunity. Our analysis results showed that KRT6B may be involved in the M2 polarization of macrophages, indicating that BLCA-derived exosomes may be involved in the regulation of macrophage differentiation. Furthermore, we found that KRT6B was associated with sensitivity to dexamethasone, acetalax and vemurafenib, and resistance to topotecan in BLCA cells. Finally, we assessed KRT6B expression in our BLCA samples and its roles in the regulation of the EMT pathway. In summary, our study demonstrated that KRT6B contained in exosomes could promote the progression of BLCA by regulating EMT and the immune response, which may be used as a prognostic marker and a target for anticancer therapy in BLCA.

## Materials and methods

### Data collection and processing

The mRNAs and proteins (n = 372) in BLCA-derived exosomes were collected from the ExoCarta database (http://www.exocarta.org/). The TCGA database (https://portal.gdc.cancer.gov/) was used to obtain transcriptome profiling data of tumours and normal tissues. Then, 19 normal samples and 414 BLCA samples were obtained. In addition, other BLCA-related datasets, including GSE13507 (n = 256) and GSE166716 (n = 24), were downloaded from the GEO database (https://www.ncbi.nlm.nih.gov/geo/). GSE13507, based on the GPL6102 platform (Illumina human-6 v2.0 expression bead chip), contains 165 primary BLCA samples, 23 recurrent nonmuscle invasive tumour tissues, 58 bladder mucosae with a normal appearance adjacent to cancer tissue, and 10 normal bladder mucosae for microarray analysis. GSE166716, based on the GPL570 platform (Affymetrix Human Genome U133 Plus 2.0 Array), contains urothelial carcinoma and matched normal urothelium samples of 12 patients. We also downloaded the matching clinical and survival data from the TCGA cohort and ultimately included 403 BLCA patients to form a training set with the TCGA data. The raw reads of the above data were processed and normalized in R software.

### Screening for differentially expressed genes

We utilized the “limma” package to screen the differentially expressed genes (DEGs) of the BLCA samples and normal samples of the TCGA dataset. A log2-fold change ≥ 1 and adjusted p value < 0.05 were considered the screening criteria. Then, we assessed the intersection of the DEGs with the exosome-encapsulated genes and obtained the exosome-related DEGs. The results were depicted in Venn diagrams, and the R package “heatmap” was used to display the DEGs.

### Survival analysis and target-gene selection

We explored the effects of the top 10 upregulated and the top 10 downregulated genes on the overall survival rate in BLCA through GEPIA (http://gepia.cancer-pku.cn/). P values < 0.05 were considered statistically significant. Ultimately, the target gene that was related to survival was determined.

### Bioinformatic analysis of the target gene

We first explored the domain of KRT6B in NCBI (https://www.ncbi.nlm.nih.gov/). Then, through the TIMER 2.0 database (http://timer.comp-genomics.org/), the expression of KRT6B in various tumours was displayed. Subsequently, we analysed the differential expression of KRT6B between normal and tumour tissues in TCGA, GEO and The Human Protein Atlas (HPA) database (https://www.proteinatlas.org/), which revealed that KRT6B was significantly increased in BLCA compared with normal urinary bladder tissue datasets, and a paired differential expression analysis was performed. Moreover, the correlations between clinical factors and KRT6B were investigated on the UALCAN website (http://ualcan.path.uab.edu/index.html). Ultimately, KRT6B, in combination with clinical factors, was subjected to both univariable and multivariable Cox regression analyses.

To explore the biological signalling pathway, gene set enrichment analysis (GSEA) was performed in the KRT6B high-expression and low-expression groups using GSEA software (v4.1.0) [8, 9]. The GO, KEGG and HALLMARK analyses were then performed. Pathways with significant enrichment results were demonstrated based on the net enrichment score (NES), gene ratio and *p* value. Gene sets with |NES| > 1, NOM *p* < 0.05, and FDR *q* < 0.25 were considered to be significant for enrichment.

The immune landscape and the relationship between KRT6B expression and 22 immune cell subtypes inferred from bulk tumour transcriptomes of BLCA patients were explored by the “CIBERSORT” algorithm [10]. TIMER 2.0 was used to comprehensively explore the profiles of tumour-infiltrating immune cells, including CD4+ T cells, CD8+ T cells, B cells, and macrophages. Furthermore, correlations between KRT6B and immune checkpoints of cancer treatment (such as CTLA4, PD-1, and PD-L1) were demonstrated.

### Drug sensitivity analysis

Containing data from 60 cancer cell lines, the NCI-60 database was analysed by the cellminer website (https://discover.nci.nih.gov/cellminer/) [11]. The expression status of target genes and *z-*score for cell sensitivity data (GI50) were downloaded from the website and assessed through Pearson correlation analysis to determine the correlation between target gene expression and drug sensitivity.

### Clinical specimens

Tumour tissues and their adjacent normal tissue were obtained from patients who were diagnosed with BLCA and had undergone surgery in the First Affiliated Hospital with Nanjing Medical University (Jiangsu Province Hospital) between 2011 and 2021. The follow-up deadline was June 2021. All patients signed informed consent before using clinical materials. The use of tissues for this study was approved by the ethics committee of the First Affiliated Hospital with Nanjing Medical University (Jiangsu Province Hospital).

### Cell culture

The BLCA cell lines (RT4, T24, BIU87, J82, UMUC3, 5637 and 253J) and the human urethral epithelial immortalized cell line (SVHUC-1 cell) were purchased from the Type Culture Collection of the Chinese Academy of Sciences (Shanghai, China) and cultured in DMEM (Gibco, Thermo Fisher Scientific, USA) containing 10% fetal bovine serum (FBS; Biological Industries, Israel) and 1% penicillin/streptomycin (Gibco, Thermo Fisher Scientific, USA). All cell lines were cultured at 37 °C in a humidified incubator containing 5% CO_2_.

### Transfection

To reduce the off-target effect of the siRNAs in T24 and BIU87 cells, three independent siRNAs targeting KRT6B and a negative control siRNA were constructed and generated by HANBIO (Shanghai, China). The targeting sequences were si-KRT6B#1 (5′-GAGGACCUCAAGAACAAAU-3′) and si-KRT6B#2 (5′-CCCUCAAGGAUGCUAAGAA-3′). The control siRNA sequence was 5′-UUCUCCGAACGUGUCACGU-3′. SiRNA transfection was performed by using Lipofectamine 3000 reagent (Invitrogen, Thermo Fisher Scientific, USA) according to the manufacturer’s protocols.

### RNA isolation and real-time quantitative PCR (qRT-PCR)

Total RNA was extracted from tissues and cell lines using TRIzol reagent (Invitrogen, Thermo Fisher Scientific, USA) following the manufacturer’s instructions. The concentration of RNA used for qRT-PCR was 500 ng/µl. And cDNA was synthesized using HiScript II (Vazyme, China). qRT-PCR for RNA analysis was performed on a StepOne Plus Real-Time PCR system (Applied Biosystems, USA). Three replicates were used for each sample, and the data were analysed by comparing CT values. The sequences of the primers, which were purchased from TSINGKE Biological Technology (Beijing, China), were listed in Additional file 1: Table S1. Fold changes in mRNA expression were calculated using the 2^−ΔΔ^CT method and normalized against β-actin with ABI Step One software version 2.1.

### Western Blotting (WB)

Total cellular proteins were lysed by RIPA buffer containing protease inhibitors (Sigma, USA). The protein extracts were harvested and quantified by bicinchoninic acid (BCA) analysis (Beyotime, China). Protein extractions were separated by 10% SDS-PAGE and transferred onto polyvinylidene fluoride (PVDF) membranes (Millipore, USA). After incubation with a high-affinity anti-KRT6B antibody (1:2000, Proteintech, USA), anti-vimentin antibody (1:1000, Proteintech, USA) or anti-GAPDH antibody (1:1000, Cell Signaling Technology, USA), the membranes were then incubated with peroxidase (HRP)-conjugated secondary antibody (1:1000, Cell Signaling Technology, USA). After washing, the signals were detected using a chemiluminescence system (Millipore, USA) and analysed using Image Lab Software (Bio-Rad, USA).

### Cell proliferation and colony formation assays

For the cell proliferation assay, cells were plated on 96-well plates at 2000 cells/well. At 24, 48, 72 and 96 h after seeding, the cells were incubated in 10 μl CCK-8 diluted in culture media at 37 °C for 1 h. The absorbance was measured at 450 nm with a microplate reader (Tecan, Switzerland).

For the colony formation assay, cells were plated on 6-well plates at 1000 cells/well for T24 cells or 800 cells/well for BIU87 cells and incubated at 37 °C in a humidified incubator for 2 weeks. After fixation with methanol, the cells were stained with 0.1% crystal violet for 30 min. Then, the colonies were imaged and counted.

### Transwell assay

Transfected cells were seeded into the upper chambers with serum-free medium, which were coated with or without Matrigel (BD Biosciences, USA) for the transwell assay. Medium containing 10% FBS was added to the bottom chambers. After incubation at 37 °C for 24 h, cells attached to the upper surface of the membrane were carefully removed with cotton swabs. Cells that reached the underside of the chamber were fixed with 10% formalin, stained with crystal violet for 15 min at room temperature and counted.

### Wound scratch assay

A wound scratch assay was carried out to determine the effect of KRT6B on cell migration ability. Briefly, a straight wound was scratched using a 200-μl pipette tip when the transfected cells reached 90–95% confluence in 6-well plates. The cells were washed with phosphate-buffered saline (PBS) to remove the detached cells and maintained at 37 °C in a humidified incubator containing 5% CO_2_. Images of the wound closure were captured at 0 and 24 h with a digital camera system (Olympus Corp, Tokyo, Japan).

### Statistical analysis

All statistical analyses were performed in R software (version 4.1.1) and GraphPad Prism (version 8.0), and a *p* < 0.05 (two-sided) was considered statistically significant. All experiments were repeated more than three times.

## Results

### Identification of exosomal DEGs in BLCA

**Fig. 1:**
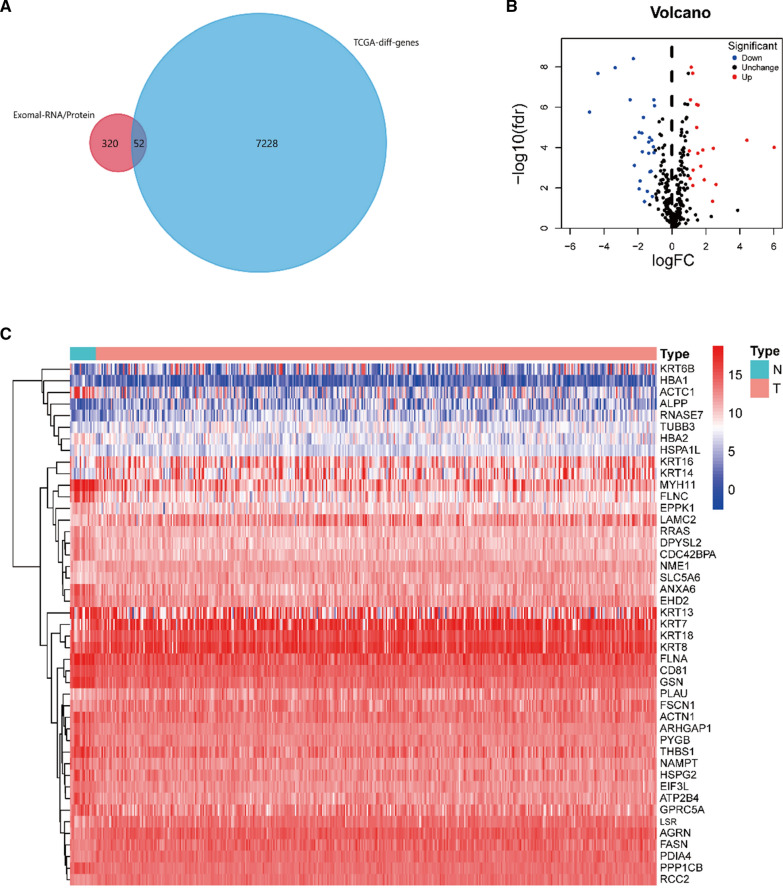
Different expression of 52 exosome-encapsuled genes in BLCA. **A** 52 differentially expressed exosome-encapsuled genes shown in venn diagram by FunRich 3.1.4 software. **B** Volcano plot of differentially expressed genes in the TCGA-BLCA cohort. The red dot represented the upregulated gene, while the blue dot represented the downregulated gene. **C** The heatmap of the differentially expressed exosome-encapsuled genes in 414 BLCA and 19 normal tissues from the TCGA database. The color bar from red to blue denotes high to low gene expression

We identified 7280 DEGs with |log2fc| > 1 and *p* value < 0.05 using the “limma” package. Fifty-two DEGs were detected in the BLCA-derived exosomes (Fig. 1A–C). The top 10 upregulated and the top 10 downregulated exosomal DEGs, according to their |log2fc|, are listed in Table 1.

**Table 1:** The top 20 differently expressed exosomal genes in TCGA

Gene symbol	Description	Normal	Tumor	logFC	P-value	FDR
UP						
ALPP	Alkaline phosphatase, placental	10.634	690.697	6.021	< 0.001	< 0.001
KRT14	Keratin 14	1487.308	31,827.192	4.419	< 0.001	< 0.001
RNASE7	Ribonuclease A family member 7	35.990	218.149	2.600	0.003	0.007
LAMC2	Laminin subunit gamma 2	2561.991	13,904.194	2.440	< 0.001	< 0.001
KRT6B	Keratin 6B	1172.249	6161.766	2.394	0.027	0.047
TUBB3	Tubulin, beta 3 class III	111.384	417.378	1.906	0.001	0.004
PLAU	Plasminogen activator,urokinase	2822.918	10,006.001	1.826	< 0.001	< 0.001
KRT7	Keratin 7	39,988.771	130,332.329	1.705	< 0.001	< 0.001
SLC5A6	Solute carrier family 5 member 6	1418.193	4135.391	1.544	< 0.001	< 0.001
KRT18	Keratin 18	19,330.929	55,592.157	1.524	< 0.001	< 0.001
Down						
ACTC1	Actin alpha cardiac muscle 1	51,994.88	1794.546	− 4.857	< 0.001	< 0.001
FLNC	Filamin C	59,376.71	2885.509	− 4.363	< 0.001	< 0.001
MYH11	Myosin, heavy polypeptide 11	237,833.6	23,446.88	− 3.342	< 0.001	< 0.001
ANXA6	Annexin A6	26,066.21	4721.63	− 2.465	< 0.001	< 0.001
GSN	Gelsolin	96,941.96	20,053.63	− 2.273	< 0.001	< 0.001
HBA1	Hemoglobin subunit alpha 1	48.22997	10.420	− 2.211	< 0.001	< 0.001
ATP2B4	ATPase plasma membrane Ca2+ transporting 4	28,496.67	6309.244	− 2.175	< 0.001	< 0.001
HSPG2	Heparan sulfate proteoglycan 2	36,948.08	9688.238	− 1.931	0.005	0.011
THBS1	Thrombospondin 1	65,492.56	17,199.6	− 1.929	< 0.001	< 0.001
PYGB	Glycogen phosphorylase B	31,392.91	8562.172	− 1.874	0.002	0.005

*TCGA* The Cancer Genome Atlas, *FDR* false discovery rate

### Survival analysis of the top dysregulated exosomal genes in BLCA

**Fig. 2:**
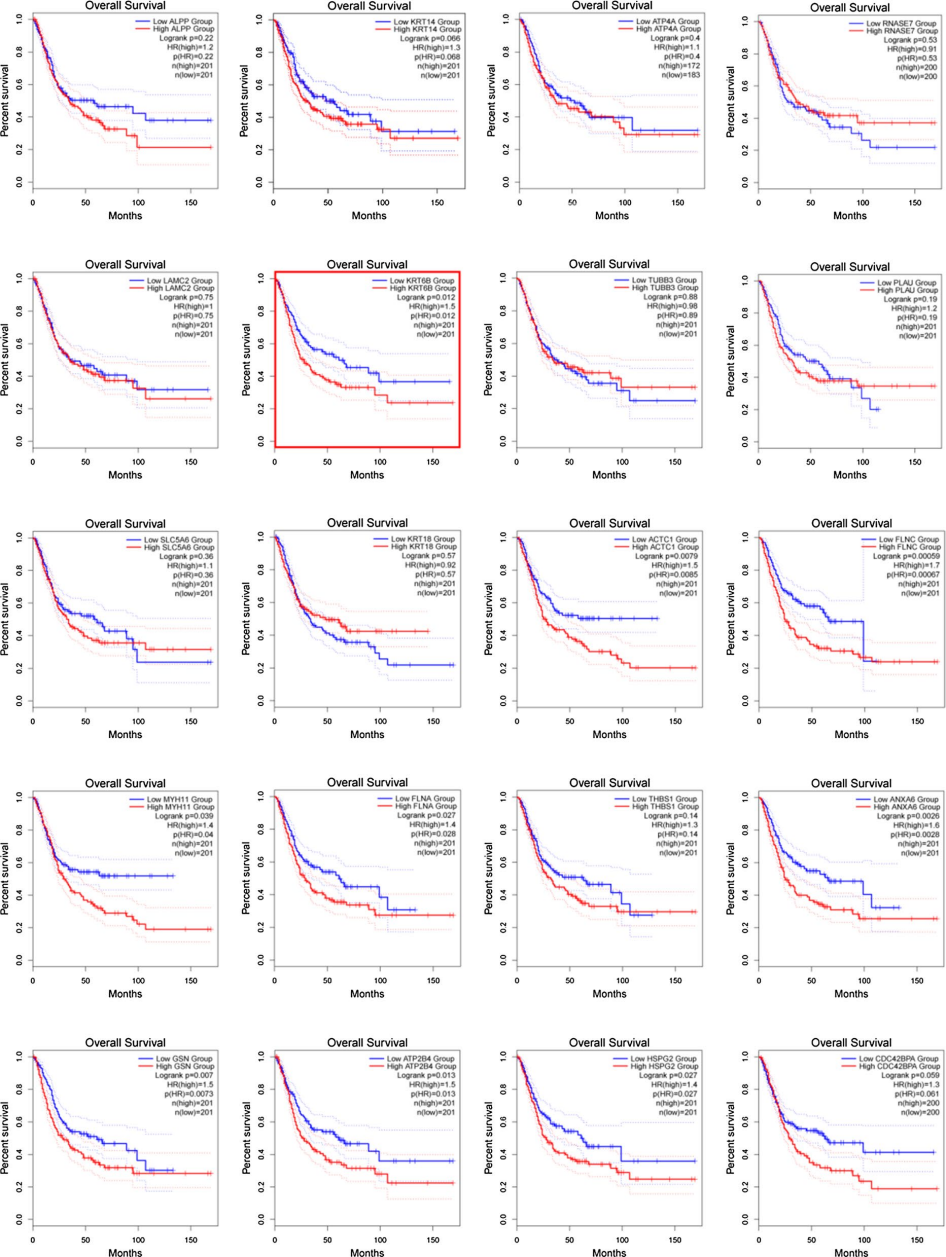
Kaplan–Meier analysis for OS of BLCA patients based on the mRNA expression of the top 10 upregulated and downregulated genes

To identify the key genes related to the prognosis of patients with BLCA, we performed survival analysis of the top 20 differentially expressed exosomal genes. The results showed that except for KRT6B, there was no statistically positive correlation between the other 9 upregulated DEGs and the survival of patients with BLCA (Fig. 2). The survival curve suggested that the high expression of KRT6B was associated with shorter overall survival (OS) and predicted a poor prognosis.

### The biological role of KRT6B in BLCA

**Fig. 3:**
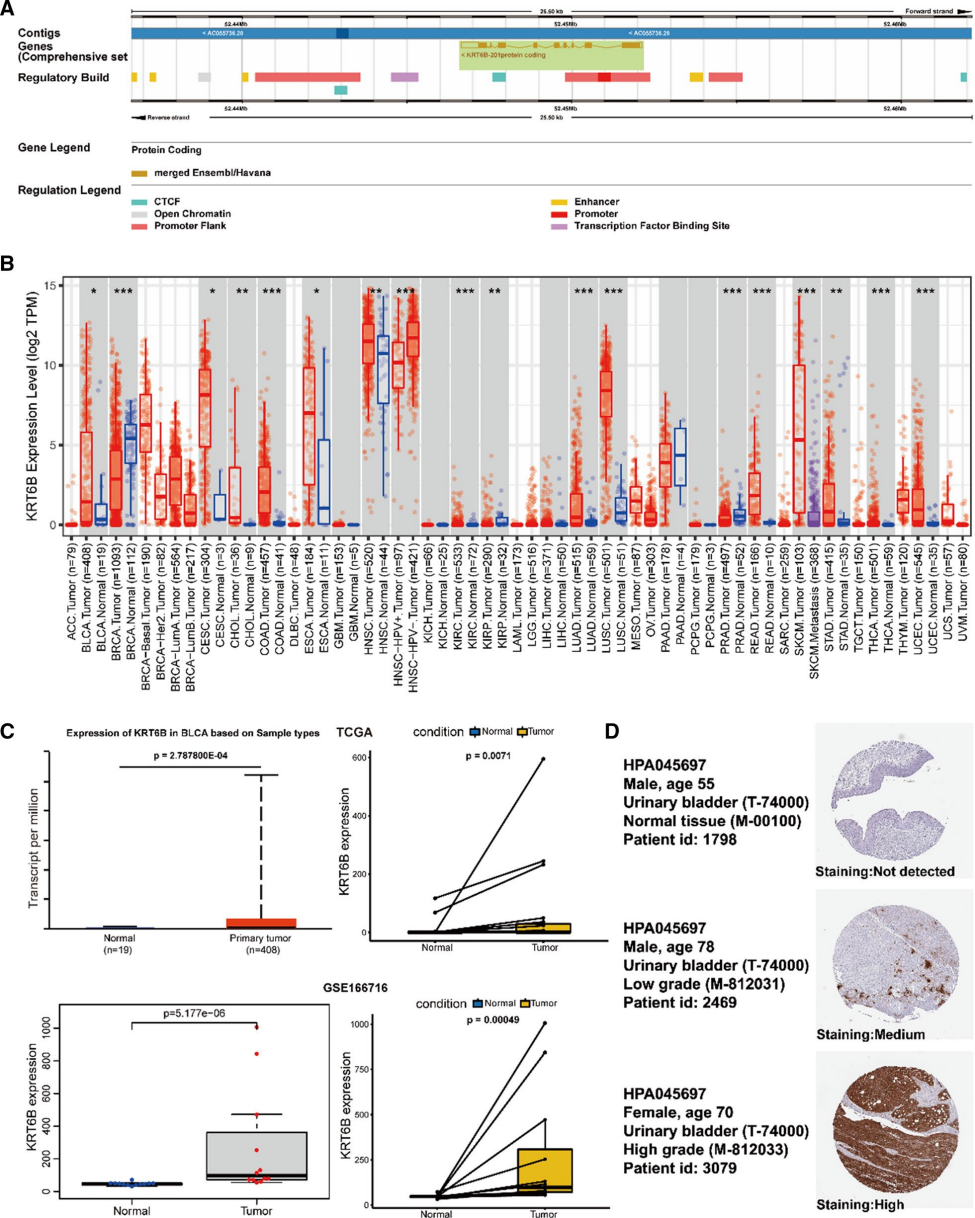
Specific generalization of KRT6B gene. **A** The domain of KRT6B gene from NCBI. **B** Differential expression of KRT6B in pan-cancer. **C** The expression of KRT6B in BLCA was higher than that in normal tissues from the TCGA and GEO datasets (GSE166716). **D** Validation of the expression of KRT6B in BLCA and normal tissues in the Human Protein Atlas (HPA) database

To further understand the biological function of KRT6B, we first queried the domain of KRT6B in the NCBI database (Fig. 3A). In addition, we analysed the differential expression of KRT6B in pancancer and matched normal tissues through the TIMER2.0 website [12, 13]. We found that KRT6B was upregulated in multiple tumours, including BLCA, CESC, CHOL, COAD, ESCA, HNSC, KIRC, LUAD, LUSC, READ, SKCM, STAD, THCA, and UCEC (Fig. 3B). The full names of the tumours and the related statistics are listed in Additional file 2: Table S2. Furthermore, the expression of KRT6B in various TDEs was obtained from the exoRBase2.0 database (http://www.exorbase.org/exoRBaseV2/toIndex). The data showed that the expression of KRT6B in various TDEs was generally higher than that in exosomes derived from normal or benign tissues (Additional file 3: Table S3). We also obtained KRT6B expression in blood and urine from the exoRBase2.0 database, and the results are provided in Additional file 4: Table S4. The TCGA and GEO datasets (GSE166716) verified that the expression of KRT6B in BLCA was higher than that in matched normal tissues (Fig. 3C). In addition, immunohistochemistry (IHC) staining datasets were retrieved from the HPA database (https://www.proteinatlas.org/), which revealed that KRT6B was significantly increased in BLCA tissue compared with normal urinary bladder tissue [14] (Fig. 3D).

**Fig. 4:**
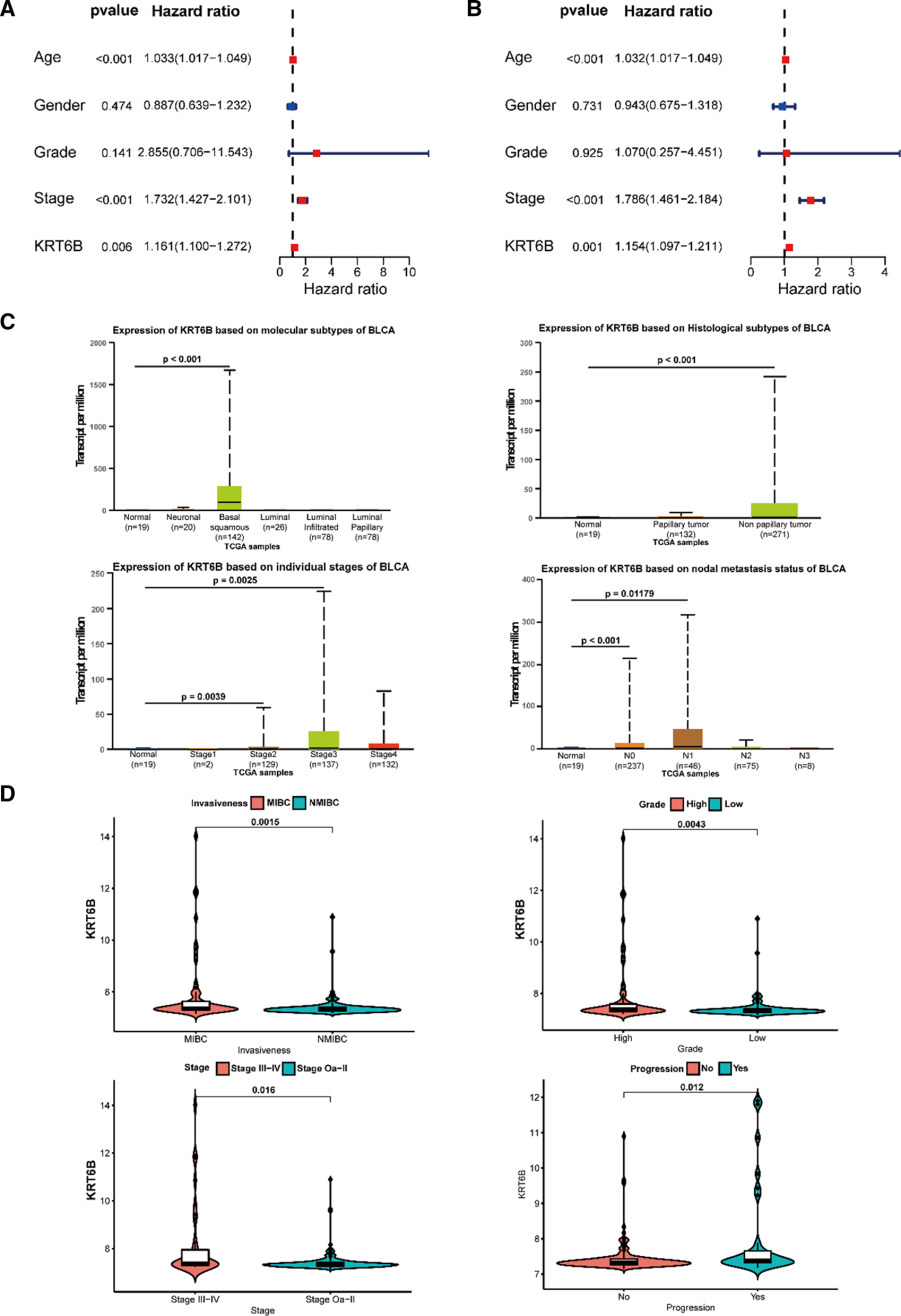
KRT6B expression was associated with clinicopathological features of BLCA based on TCGA. **A**, **B** Univariate (**A**) and multivariate (**B**) Cox analysis of KRT6B expression and clinicopathological variables. **C** KRT6B expression associated with different molecular subtypes, histological subtypes, lymph node metastasis, and higher stage of BLCA based on TCGA. **D** KRT6B expression associated with MIBC, higher grade, higher stage, and progression of BLCA based on GSE13507

Based on the expression level of KRT6B, we divided clinical data from TCGA into high- and low-expression groups (Table 2). To explore the relationship between KRT6B expression and the clinical features of BLCA, we carried out univariate Cox regression analysis and multivariate Cox regression analysis. The results indicated that the expression of KRT6B, age, and stage were independent prognostic risk factors for BLCA (Fig. 4A, B). We also found that KRT6B was variously expressed in patients with different histological subtypes, molecular subtypes, metastasis statuses, and individual cancer stages of BLCA through the UALCAN website [15] (Fig. 4C). Consistent with TCGA results, the GSE13507 data results showed that upregulated KRT6B expression was correlated with clinicopathological features in BLCA (Fig. 4D). This evidence suggests that KRT6B may be involved in the progression of BLCA, especially the transformation from noninvasive to invasive tumours.

**Table 2:** Clinicopathological features between two groups of KRT6B

Covariates	High KRT6B	Low KRT6B	P value
	No. (%)	No. (%)	
Age (years)			0.9484
< 65	74 (36.82%)	76 (37.62%)	
≥ 65	127 (63.18%)	126 (62.38%)	
Gender			0.0382
Female	62 (30.85%)	43 (21.29%)	
Male	139 (69.15%)	159 (78.71%)	
Status			0.0031
Alive	103 (51.24%)	73 (36.14%)	
Dead	98 (48.76%)	129 (63.86%)	
Grade			0.0027
High	198 (98.51%)	182 (90.1%)	
Low	3 (1.49%)	17 (8.42%)	
Unknown	0 (0%)	3 (1.49%)	
T classification			0.3839
T0	0 (0%)	1 (0.5%)	
T1	0 (0%)	3 (1.49%)	
T2	57 (28.36%)	60 (29.7%)	
T3	98 (48.76%)	94 (46.53%)	
T4	29 (14.43%)	28 (13.86%)	
Unknown	17 (8.46%)	16 (7.92%)	
N classification			0.0099
N0	124 (61.69%)	111 (54.95%)	
N1	27 (13.43%)	17 (8.42%)	
N2	28 (13.93%)	47 (23.27%)	
N3	1 (0.5%)	6 (2.97%)	
Unknown	21 (10.45%)	21 (10.4%)	
M classification			1
M0	95 (47.26%)	100 (49.5%)	
M1	5 (2.49%)	6 (2.97%)	
Unknown	101 (50.25%)	96 (47.52%)	
TNM stage			0.3435
I	0 (0%)	2 (0.99%)	
II	64 (31.84%)	64 (31.68%)	
III	75 (37.31%)	65 (32.18%)	
IV	61 (30.35%)	70 (34.65%)	
Unknown	1 (0.5%)	1 (0.5%)

**Fig. 5:**
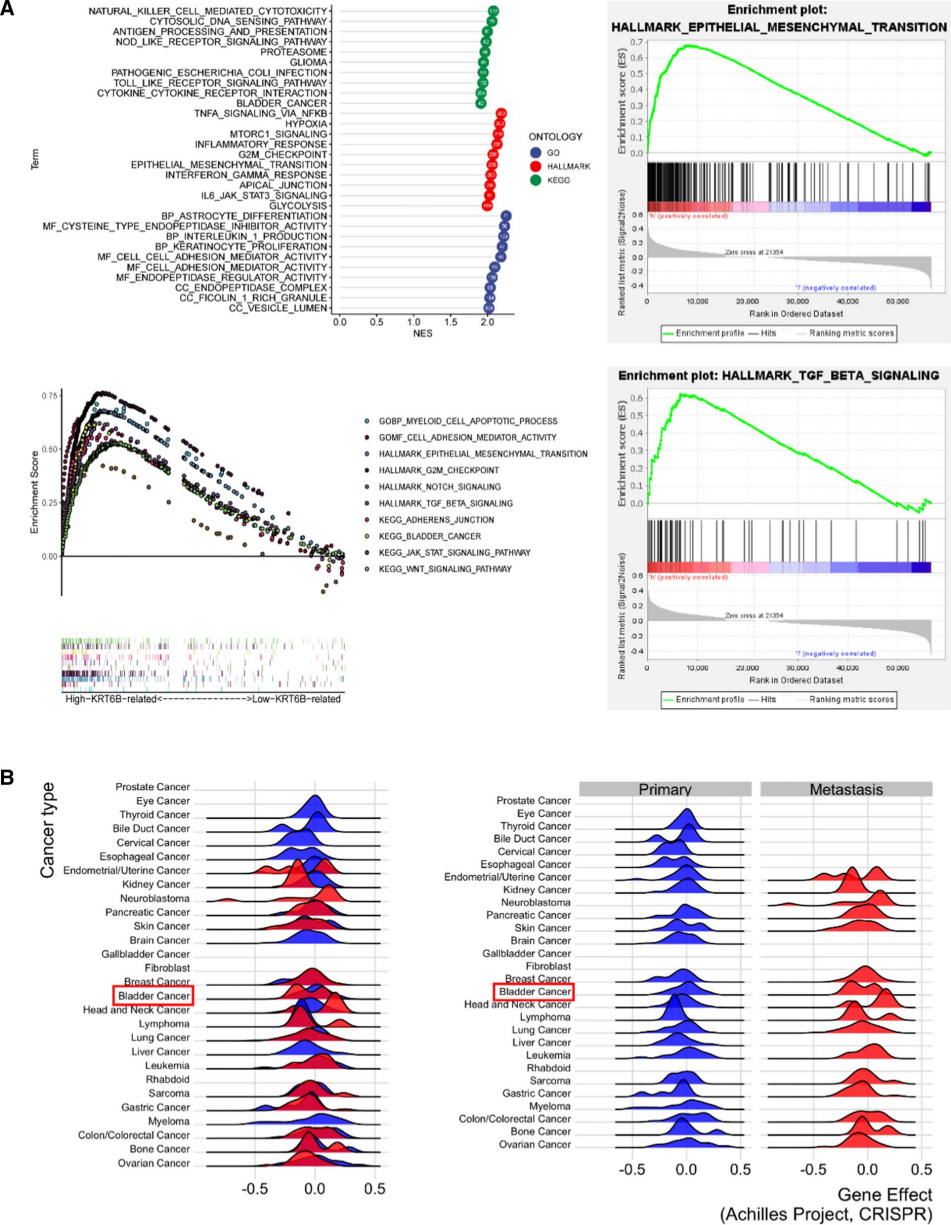
KRT6B expression correlated with EMT and immune signatures in BLCA. **A** GSEA enrichment based on TCGA-BLCA samples. **B** KRT6B expression correlated with metastasis of BLCA based on EMTome database. All gene sets were significantly enriched at nominal *p* value < 0.05 and FDR *q* value < 0.05. NES, normalized enrichment score

To clarify the mechanism of KRT6B in the development of BLCA, we conducted GSEA based on high-KRT6B expression and low-KRT6B expression groups of BLCA samples from TCGA database. The GSEA results showed that the high-KRT6B expression group was enriched in the inflammatory response pathway, including the IL-6/JAK/STAT3 signalling pathway and the interferon-γ (IFN-γ) response pathway (Fig. 5A). The results also showed that the activation of the EMT-related pathway was positively correlated with the expression of KRT6B (Fig. 5A). To verify this conclusion, we performed the same analysis on the GSE13507 dataset. The final result was consistent with that of the TCGA data (Additional file 6: Fig. S1A). In addition, analysis of the EMTome database (http://www.emtome.org/), a resource for pancancer analysis of EMT genes and signatures, showed that KRT6B was closely related to metastasis [16] (Fig. 5B, Additional file 6: Fig. S1B).

**Fig. 6:**
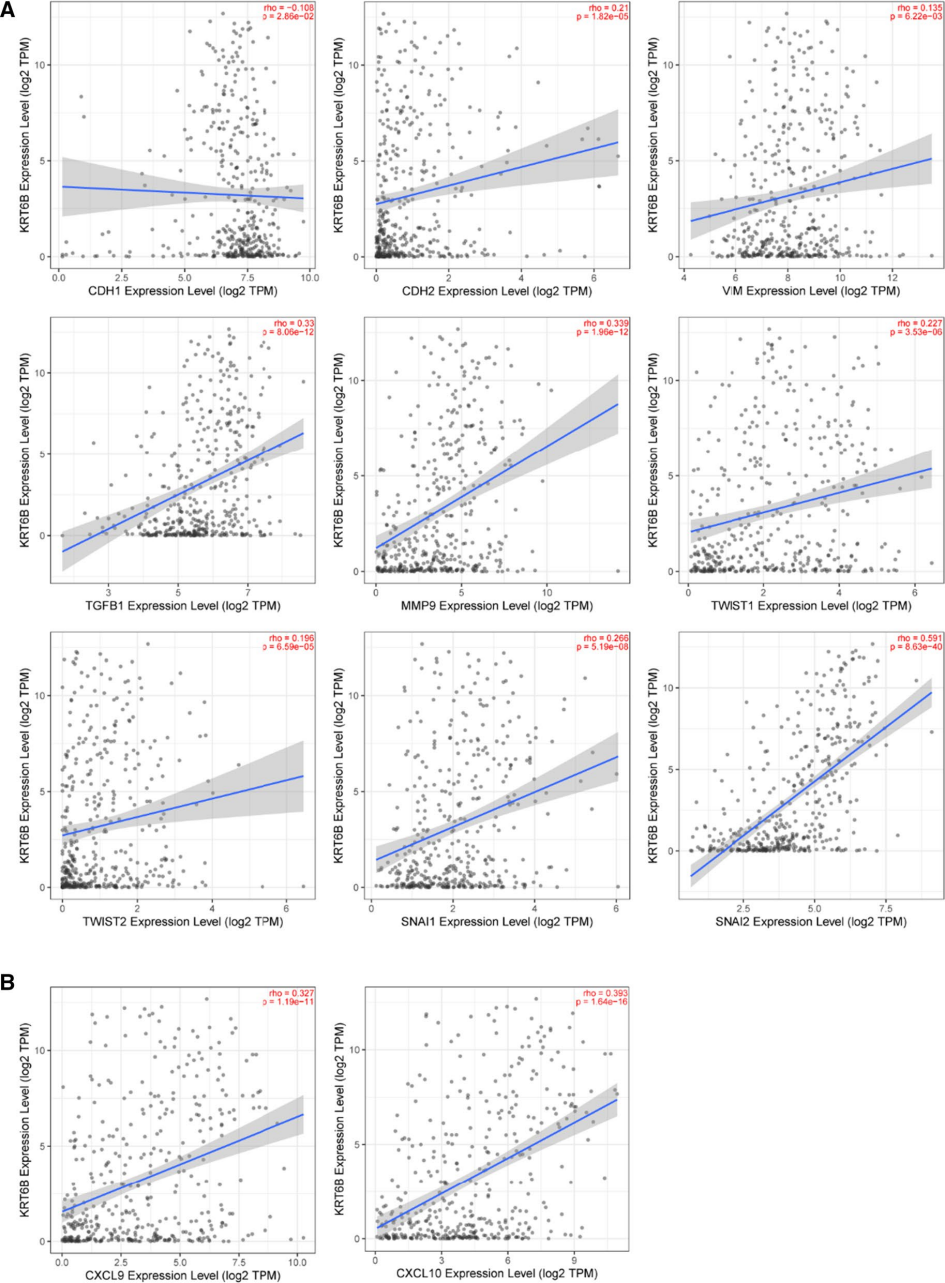
KRT6B expression was correlated with markers of EMT and immune response. **A** KRT6B expression was positively correlated with EMT-related genes, including CHD-2 (N-CAD), vimentin, TGF-β, MMP9, Twist-1, Twist-2, snail-1, and snail-2, negatively correlated with CHD-1 (E-CAD) expression. **B** KRT6B expression was positively correlated with immune-related genes, including CXCL9 and CXCL10

EMT is a key step for epithelial cells to gain invasiveness and an important condition for myometrial invasion and metastasis of BLCA cells [17, 18]. Currently, several genes have been proven to be biomarkers of the EMT process, including CDH1, CDH2, vimentin, MMP9, Twist, snail and TGF-β [19, 20]. Next, we explored the correlation between KRT6B and these biomarkers through the TIMER2.0 website. Interestingly, the results showed that there was a significant correlation between KRT6B and these key genes (Fig. 6A). This result indicated that KRT6B may participate in the EMT process of BLCA and promote the metastasis of BLCA. Additionally, the immune response and immune microenvironment have recently become hot topics in the field of cancer due to their role in regulating tumour progression [21, 22]. Our results showed that KRT6B was significantly positively correlated with immune response genes, such as CXCL9 and CXCL10 (Fig. 6B). All of these results outlined above proved that KRT6B was involved in the EMT process and immune regulation of BLCA.

**Fig. 7:**
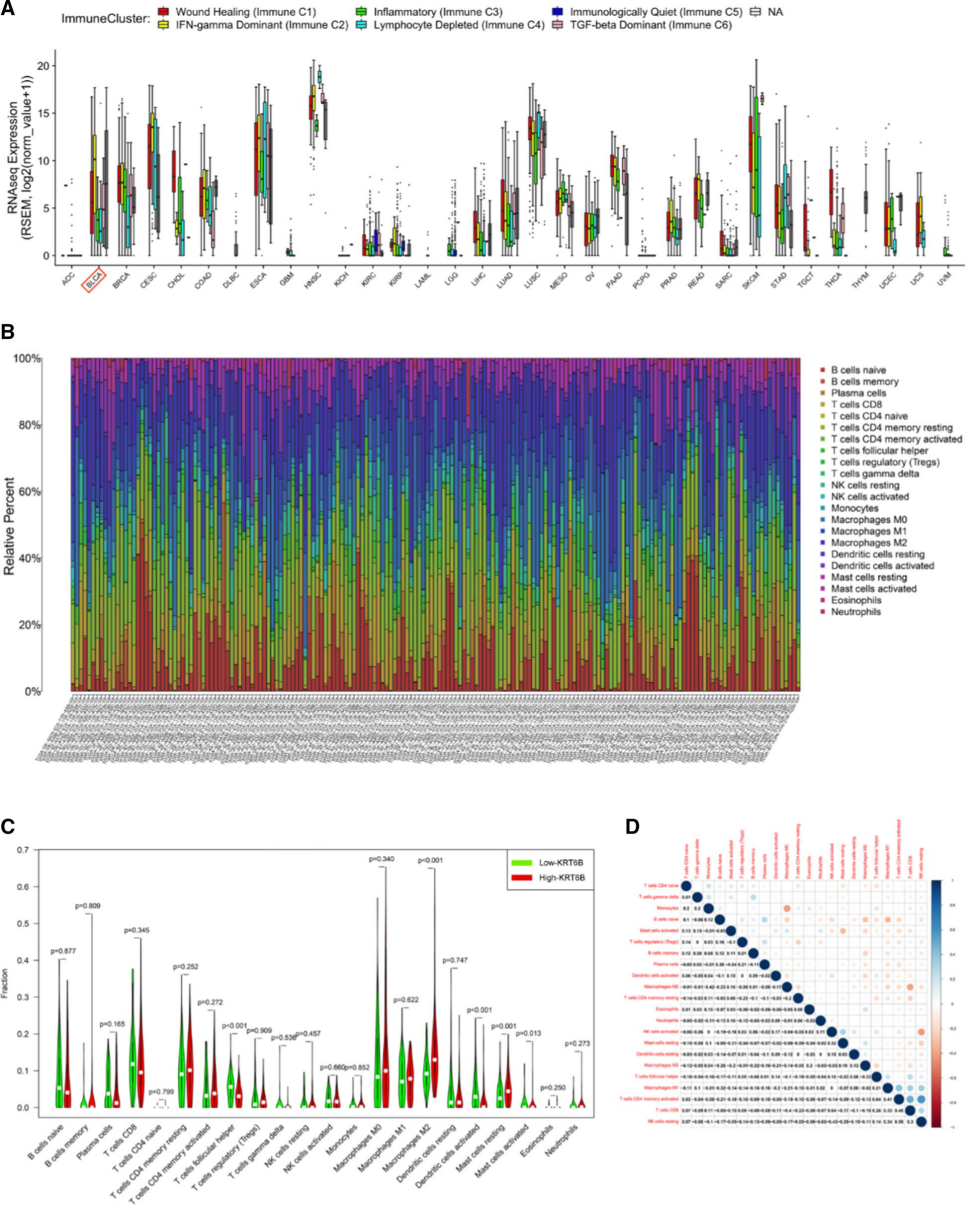
KRT6B regulated the infiltration and distribution of immune cells in BLCA. **A** Immune clusters in pan-cancer. **B** The immune landscape in BLCA based on TCGA. **C** Wilcoxon ranksum test accurately compared the difference and indicated that several immune cells conferred significantly lower infiltrating density in high KRT6B groups, including M2 macrophage (*p* < 0.001) and mast cells resting (*p* = 0.001). **D** Heatmap of 22 immune infiltration cells’ correlation in tumor samples of TCGA cohort

To explore the potential role of KRT6B in the tumour microenvironment of BLCA, we first utilized the EMTome database and CIBERSORT algorithm to show the landscape of immune cell infiltration in BLCA from TCGA data (Fig. 7A, B). According to the expression of KRT6B, we divided the samples into two groups and investigated the different distributions of immune cells between them. We found that several immune cells conferred significantly higher infiltrating density in the high KRT6B group, including M2 macrophages (*p* < 0.001), resting mast cells (*p* = 0.001) and activated mast cells (*p* = 0.013) (Fig. 7C). A heatmap showing the correlation of 22 immune infiltrating cells with tumour samples from the TCGA cohort was shown (Fig. 7D).

**Fig. 8:**
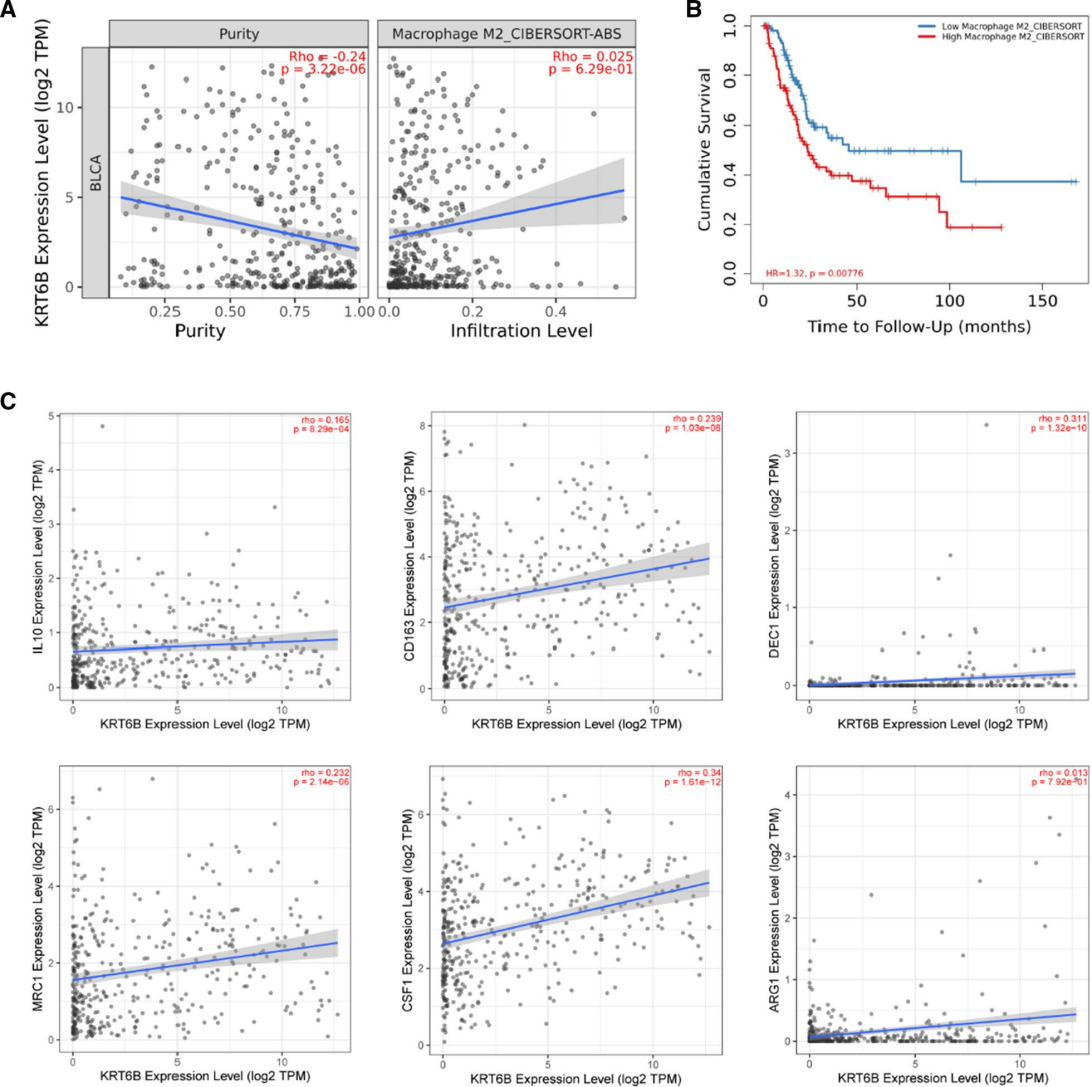
KRT6B significantly correlated with M2 Macrophage infiltration in BLCA. **A** Correlations between KRT6B expression and M2 Macrophage infiltration level. **B** Levels of M2 Macrophage infiltration and overall survival based on data obtained from TIMER 2.0. **C** Positive correlations between KRT6B expression and markers of M2 Macrophage, including IL-10, CD163, DEC1, MRC1, CSF1 and ARG1

Correlation analysis from TIMER 2.0 revealed that the infiltration level of M2 macrophages had a positive association with the expression of KRT6B (Fig. 8A). In addition, K–M survival analyses showed that patients with higher M2 macrophage infiltration had poor survival [23, 24] (Fig. 8B). The above evidence suggested that KRT6B had a certain relationship with the different distributions of immune cells, especially the polarization of M2 macrophages in BLCA. To verify this hypothesis, we individually analysed the correlation of M2 macrophages and their key markers with KRT6B. Ultimately, we concluded that the KRT6B expression level was positively correlated with the infiltration level of M2 macrophages (Fig. 8C).

**Fig. 9:**
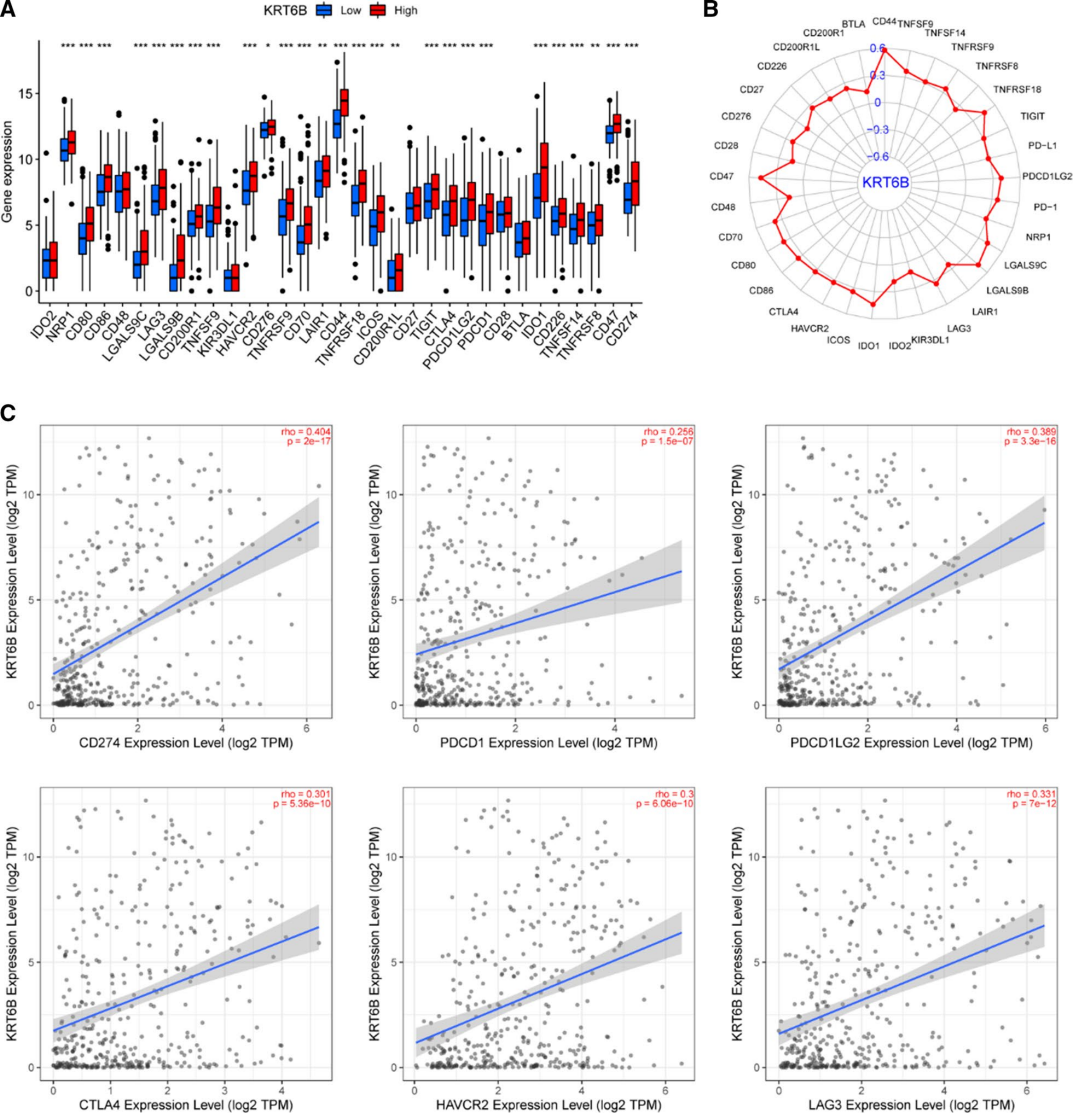
KRT6B expression was significantly associated with immune checkpoints. **A** Box plot of immune checkpoints based on KRT6B expression. **B** Radar plot of the correlation between immune checkpoints and KRT6B expression. **C** The correlation between KRT6B expression and some typical immune checkpoints based on TIMER 2.0

Currently, immune checkpoint inhibitors are widely studied and have achieved unprecedented success in cancer immunotherapy [25, 26]. In our study, we found that multiple checkpoint genes showed differential expression in BLCA (Fig. 9A). Given that KRT6B was tightly linked to immunity, we further investigated the correlation between KRT6B and immune checkpoint genes, such as PD-L1. Correlations between KRT6B and immune checkpoints were observed (Fig. 9B, C). All of the above results suggested that BLCA patients with high KRT6B expression may benefit more from therapy combined with immune checkpoint blockade (ICB).

Finally, the influence of target genes on drug sensitivity was assessed using the CellMiner database, which could facilitate better precision treatment. Drug sensitivity was measured by *z-*score, and the higher scores implied that cells were more sensitive to the drug treatment (Additional file 7: Fig. S2). We ultimately found that KRT6B was associated with increased sensitivity of BLCA to dexamethasone, acetalax and vemurafenib, and resistance to topotecan treatment of BLCA cells. These findings provided a new alternative strategy for the precise treatment of BLCA patients.

### KRT6B expression in our BLCA samples regulated the EMT signalling pathway

**Fig. 10:**
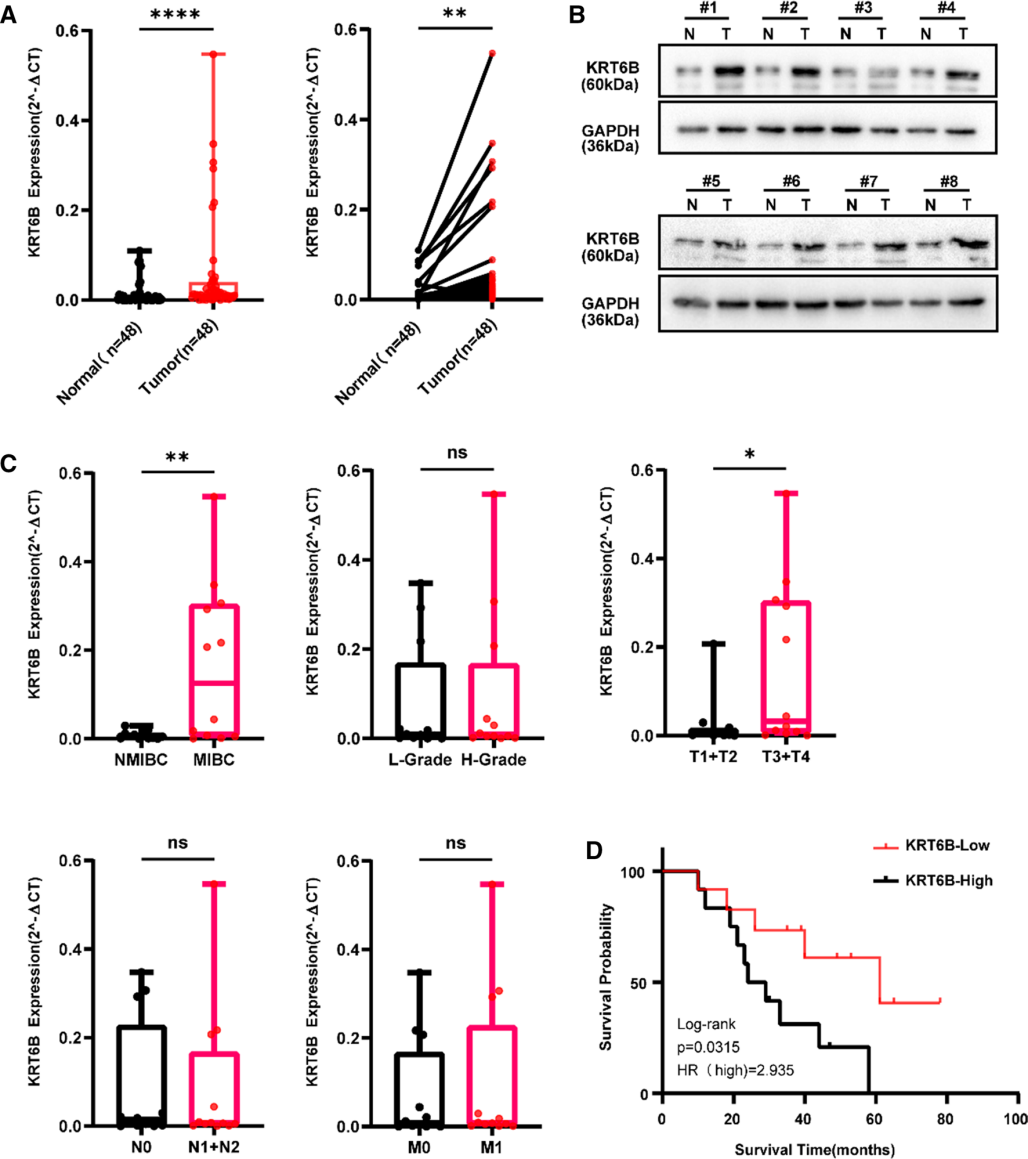
Elevated levels of KRT6B in BLCA were correlated with poor prognosis. **A** Expression of KRT6B in 48 paired BLCA tissues and normal bladder tissues was evaluated by qRT-PCR. **B** 8 pairs of BLCA tissues and adjacent normal tissues were estimated by WB. **C** The relationship between KRT6B expression and myometrial invasion, stage, and clinical grade of BLCA. **D** The overall survival of 24 patients with KRT6B^high^ or KRT6B^low^ BLCA was analyzed by the log-rank test

To further explore the expression of KRT6B in BLCA clinical samples, qRT-PCR and WB were conducted on 48 pairs of BLCA tissue samples and matched adjacent normal tissue samples. As shown in Fig. 10A, the expression of KRT6B was higher in BLCA tissue samples than in adjacent normal tissue samples according to the qRT-PCR results (Fig. 10A, *p* < 0.01). Consistently, the protein level and the corresponding mRNA level showed the same result (Fig. 10B, Additional file 5: Table S5). In addition, the relationship between the expression of KRT6B and the clinicopathological characteristics of BLCA was assessed. The results showed that high expression of KRT6B was positively associated with tumour invasiveness and T stage of BLCA, suggesting that KRT6B played an essential role in the progression of BLCA. Other features, including N stage, M stage, and tumour grade, were not significantly associated with KRT6B expression (Fig. 10C, *p* > 0.05). Furthermore, high expression of KRT6B was shown to be associated with poor OS in BLCA patients (Fig. 10D, *p* < 0.05).

**Fig. 11:**
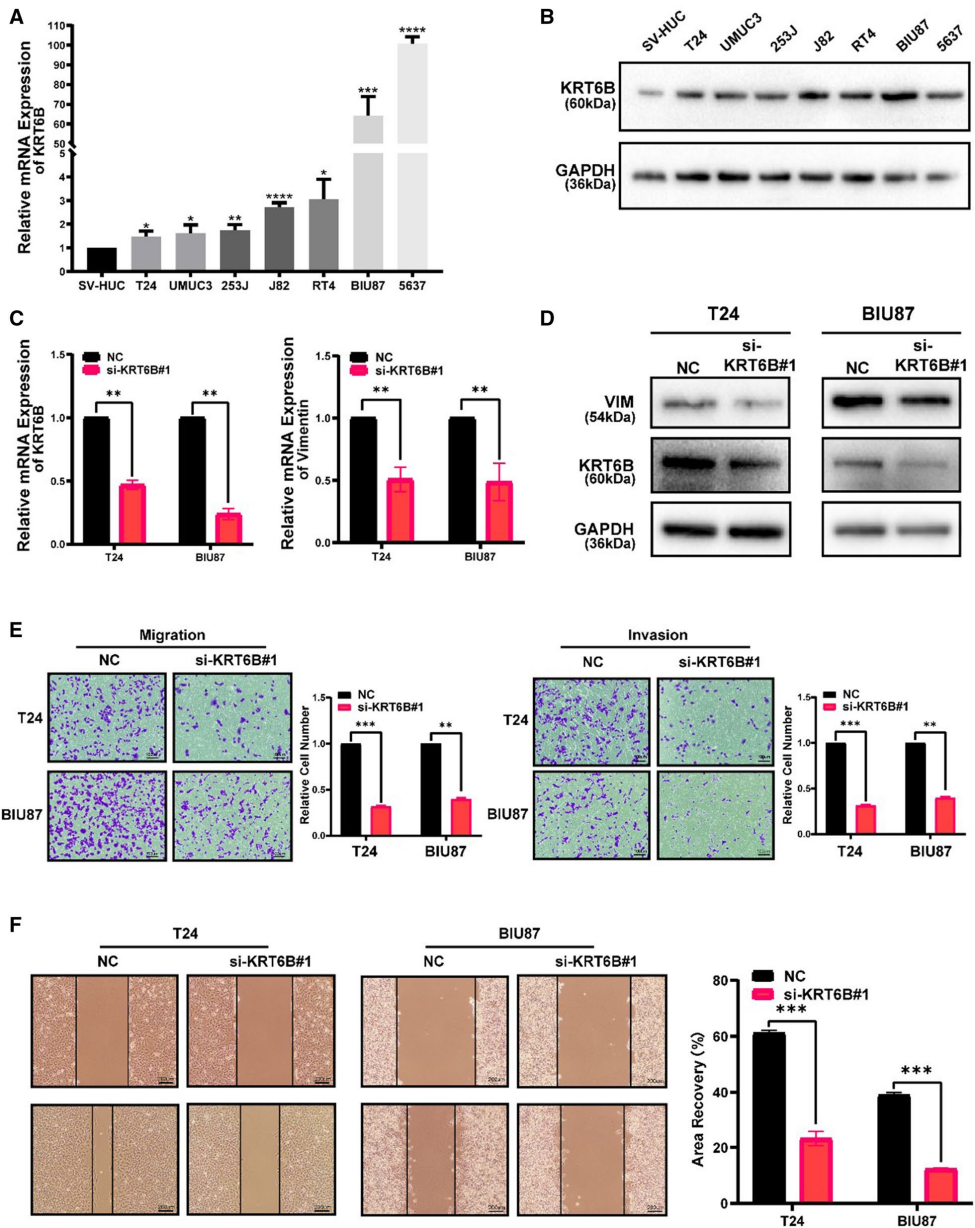
KRT6B promoted BLCA cell migration and invasion. **A** mRNA level of KRT6B in the human ureteral epithelial immortalized cell line and the BLCA cell lines. **B** Protein level of KRT6B in the human ureteral epithelial immortalized cell line and the BLCA cell lines. **C** KRT6B mRNA expression were decreased in T24 and BIU87 after transfected with KRT6B siRNA, and positively correlated with vimentin. **D** The protein level of KRT6B were decreased in T24 and BIU87 after transfected with KRT6B siRNA, and positively correlated with vimentin. **E** Transwell migration assays and invasion assays showed that KRT6B knockdown suppressed migration and invasion in T24 and BIU87 cells. **F:** Wound healing assays showed that KRT6B knockdown suppressed migration in T24 and BIU87 cells (original magnification, ×200)

To further investigate the role of KRT6B, we verified the expression of KRT6B in a human urethral epithelial immortalized cell line and the BLCA cell lines through qRT-PCR and WB (Fig. 11A, B, *p* < 0.05). We found that the expression of KRT6B in the BLCA cell lines was generally higher than that in the human urethral epithelial immortalized cell line. Then, we knocked down KRT6B in both T24 and BIU87 cells by specifically targeting KRT6B with siRNA. The KRT6B mRNA and protein levels were significantly downregulated compared with those in the control group (Fig. 11C, D, *p* < 0.05). The qRT-PCR and WB results also showed that knockdown of KRT6B resulted in a significant reduction in both the mRNA and protein levels of the mesenchymal marker vimentin, suggesting that KRT6B was involved in the EMT pathway of BLCA (Fig. 11C, D, *p* < 0.05). Transwell migration assays and invasion assays showed that KRT6B knockdown suppressed migration and invasion in T24 and BIU87 cells (Fig. 11E, *p* < 0.05). In addition, wound healing assays showed that the wound scratch closed up much more slowly when KRT6B was knocked down (Fig. 11F, *p* < 0.05). In addition, the CCK8 assay and the colony formation assay indicated that KRT6B could enhance the proliferation of BLCA cells (Additional file 8: Fig. S3, *p* < 0.05). Together, these data suggested that KRT6B plays an important role in the migration and invasion of BLCA cells.

## Discussion

Bladder cancer is a common malignant tumour that has a high progression rate and seriously affects people’s lives [27]. Early diagnosis and timely treatment can improve the survival rate and effectively avoid invasion and distant metastasis, which is the key factor in improving the prognosis of patients.

Exosomes are specific EVs with a variety of biological functions that exist widely in a variety of biological body fluids and function in intercellular communication through the proteins, nucleic acids, lipids and metabolites they carry [28]. In addition, exosomes are crucial for understanding the mechanisms associated with the development, metastasis and drug resistance of BLCA [29]. A large number of studies have shown that exosomes are important carriers of genetic material in the TME and communicate with tumour cells or surrounding normal tissues. The proteins, nucleic acids and other contents carried and released by EVs can change the TME and promote EMT, angiogenesis, tumour immune escape, and the formation of the premetastatic niche (PMN), thus promoting tumour growth, invasion and metastasis of BLCA [30]. As vital factors in the progression of BLCA, exosomes are promising noninvasive biomarkers for the clinical diagnosis and treatment of BLCA.

In this study, mRNAs and proteins in BLCA-derived exosomes were collected from the ExoCarta database and analysed comprehensively in combination with the TCGA database and GEO database. The results of the differential analysis showed that KRT6B was one of the top 10 upregulated genes in BLCA. Subsequent survival analysis further demonstrated that KRT6B was correlated with the OS of patients with BLCA. Then, univariate and multivariate Cox regression analyses performed in the whole cohort of TCGA and GEO indicated that age, stage, and KRT6B were significantly associated with the OS of BLCA. To explore the correlation between KRT6B and the clinicopathological features of BLCA, Pearson correlation was used. As expected, the expression of KRT6B was positively related to the histological subtypes, stages, metastasis and myometrial invasion of BLCA. Taken together, the above results suggested that clinical outcomes were worse in patients with high KRT6B expression than in those with low KRT6B expression.

To further explore the role of KRT6B in BLCA, we carried out GSEA on the functions and pathways that may be involved in BLCA. The results of the analysis showed that KRT6B was significantly related to EMT and immune mechanisms, especially the polarization of M2 macrophages. At the same time, the analysis results from the EMTome database confirmed the importance of KRT6B in BLCA metastasis. In previous studies, exosomes were reported to be widely involved in the tumorigenesis and development of BLCA, including the regulation of the immune microenvironment and EMT process of BLCA [31, 32]. Consistent with these results, our experimental results showed that KRT6B knockdown significantly inhibited the invasion and migration of BLCA cells. These results suggested that KRT6B could promote BLCA progression by regulating EMT. However, the molecular mechanism by which KRT6B regulates EMT in BLCA development still needs to be further studied.

In our study, we found that KRT6B was involved in regulating EMT and tumour immunity in BLCA. Since KRT6B can be detected in BLCA-derived exosomes, we hypothesized that TDEs could regulate the EMT process and immunity by transporting encapsulated KRT6B to adjacent cells and the surrounding microenvironment in BLCA.

There are still several limitations to our study. First, whether KRT6B regulates EMT and the immune response directly or indirectly through exosomes needs to be further studied. Second, the function of migration and invasion in vitro still needs to be verified in an animal metastasis model. Finally, our findings are just proof-of-concept, which requires more experiments and studies for confirmation.

## Conclusions

In summary, our study indicated that KRT6B was involved in the progression of BLCA through EMT and the immune response, which implied that KRT6B in BLCA-derived exosomes could be a prognostic marker for BLCA and a new candidate target for BLCA therapy.

## Supplementary Information

**Additional file 1: Table S1.** The primer sequences in our study.

**Additional file 2: Table S2.** The abbreviations, sample numbers and full names of various tumors in TCGA database.

**Additional file 3: Table S3.** The expression of KRT6B in various TDEs.

**Additional file 4: Table S4.** The KRT6B expression in blood and urine.

**Additional file 5: Table S5.** Mean expression level of KRT6B.

**Additional file 6: Figure S1.** KRT6B expression correlated with EMT and immune signatures in BLCA. **A** GSEA enrichment based on GSE13507 samples. **B** KRT6B expression correlated with metastasis of BLCA based on EMTome database. All gene sets were significantly enriched at nominal *p* value < 0.05 and FDR *q* value < 0.05. NES, normalized enrichment score.

**Additional file 7: Figure S2.** The scatter plot indicates the correlation between KRT6B expression and drug sensitivity (the *z*-score of the CellMiner interface) for the Pearson correlation test using NCI-60 cell line data.

**Additional file 8: Figure S3.** KRT6B enhanced the proliferation of BLCA cells. **A** Colony formation assay indicated that KRT6B could enhance the proliferation of BLCA cells. **B** CCK8 assay indicated that KRT6B could enhance the proliferation of BLCA cells.

## Data Availability

The original contributions presented in the study are included in the article/supplementary material. Further inquiries can be directed to the corresponding authors.
