# Supplementary material for: Bladder cancer-derived exosomal KRT6B promotes invasion and metastasis by inducing EMT and regulating the immune microenvironment
Source: J Transl Med. 2022 Jul 6;20:308. doi: 10.1186/s12967-022-03508-2 (PMC9258227; doi:10.1186/s12967-022-03508-2)
Supplement: Supplementary file 1 — Additional file 1: Table S1. The primer sequences in our study. [file 12967_2022_3508_MOESM1_ESM.docx]

**Table S1: The primer sequences in our study**

| Gene | Primer sequences |
| --- | --- |
| β-Actin |  |
| Forward | AGCGAGCATCCCCCAAAGTT |
| Reverse | GGGCACGAAGGCTCATCATT |
| KRT6B |  |
| Forward | TCAGCACTCAGACATGCGAA |
| Reverse | TGCAGCTGGACCTAGACTGA |
| Vimentin |  |
| Forward | AAAAGTCCGCACATTCGAGC |
| Reverse | CGCTGCTAGTTCTCA-GTGCT |
