# Supplementary material for: Bladder cancer-derived exosomal KRT6B promotes invasion and metastasis by inducing EMT and regulating the immune microenvironment
Source: J Transl Med. 2022 Jul 6;20:308. doi: 10.1186/s12967-022-03508-2 (PMC9258227; doi:10.1186/s12967-022-03508-2)
Supplement: Supplementary file 2 — Additional file 2: Table S2. The abbreviations, sample numbers and full names of various tumors in TCGA database. [file 12967_2022_3508_MOESM2_ESM.docx]

**Table S2: TCGA Datasets Evaluated**

| **Symbol** | **N** | **Name** |
| --- | --- | --- |
| ACC | 79 | Adrenocortical carcinoma |
| BLCA | 427 | Bladder urothelial carcinoma |
| BRCA | 1205 | Breast invasive carcinoma |
| CESC | 307 | Cervical squamous cell carcinoma and endocervical adenocarcinoma |
| CHOL | 45 | Cholangiocarcinoma |
| COAD | 498 | Colon adenocarcinoma |
| DLBC | 48 | Lymphoid neoplasm diffuse large B-cell lymphoma |
| ESCA | 195 | Esophageal carcinoma |
| GBM | 158 | Glioblastoma multiforme |
| HNSC | 564 | Head and neck squamous cell carcinoma |
| KICH | 91 | Kidney chromophobe |
| KIRC | 605 | Kidney renal clear cell carcinoma |
| KIRP | 322 | Kidney renal papillary cell carcinoma |
| LAML | 173 | Acute myeloid leukemia |
| LGG | 516 | Brain lower grade glioma |
| LIHC | 421 | Liver hepatocellular carcinoma |
| LUAD | 574 | Lung adenocarcinoma |
| LUSC | 552 | Lung squamous cell carcinoma |
| MESO | 87 | Mesothelioma |
| OV | 303 | Ovarian serous cystadenocarcinoma |
| PAAD | 182 | Pancreatic adenocarcinoma |
| PCPG | 182 | Pheochromocytoma and paraganglioma |
| PRAD | 549 | Prostate adenocarcinoma |
| READ | 176 | Prostate adenocarcinoma |
| SARC | 259 | Sarcoma |
| SKCM | 471 | Skin cutaneous melanoma |
| STAD | 450 | Stomach adenocarcinoma |
| TGCT | 150 | Testicular germ cell tumors |
| THCA | 560 | Thyroid carcinoma |
| THYM | 120 | Thymoma |
| UCEC | 580 | Uterine Corpus Endometrial Carcinoma |
| UCS | 57 | Uterine carcinosarcoma |
| UVM | 80 | Uveal melanoma |
