# Supplementary figures and images for: Bladder cancer-derived exosomal KRT6B promotes invasion and metastasis by inducing EMT and regulating the immune microenvironment
Source: J Transl Med. 2022 Jul 6;20:308. doi: 10.1186/s12967-022-03508-2 (PMC9258227; doi:10.1186/s12967-022-03508-2)

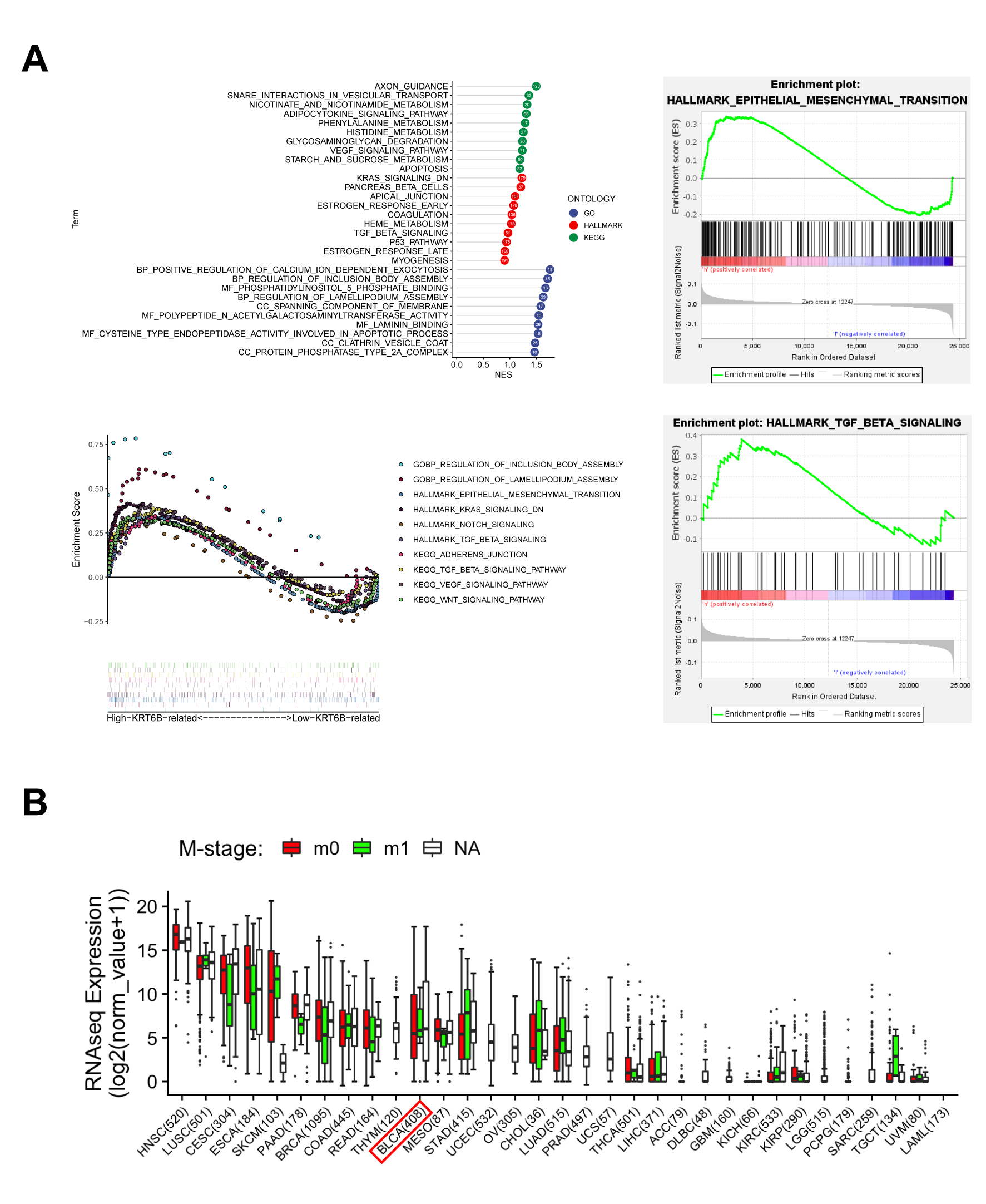

Supplement: Supplementary file 6 — Additional file 6: Figure S1. KRT6B expression correlated with EMT and immune signatures in BLCA. A GSEA enrichment based on GSE13507 samples. B KRT6B expression correlated with metastasis of BLCA based on EMTome database. All gene sets were significantly enriched at nominal p value < 0.05 and FDR q value < 0.05. NES, normalized enrichment score. [file 12967_2022_3508_MOESM6_ESM.tif]

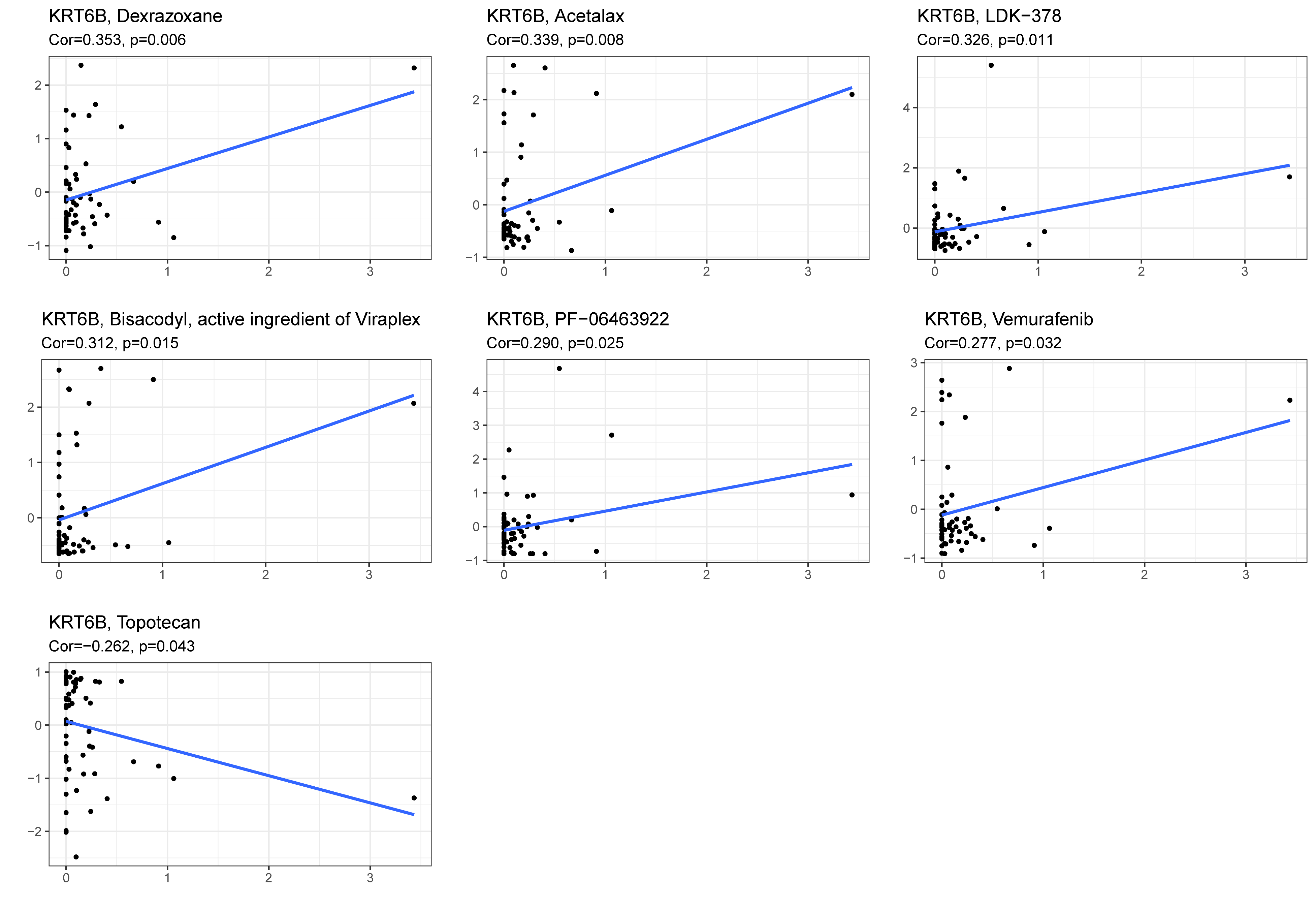

Supplement: Supplementary file 7 — Additional file 7: Figure S2. The scatter plot indicates the correlation between KRT6B expression and drug sensitivity (the z-score of the CellMiner interface) for the Pearson correlation test using NCI-60 cell line data. [file 12967_2022_3508_MOESM7_ESM.tif]

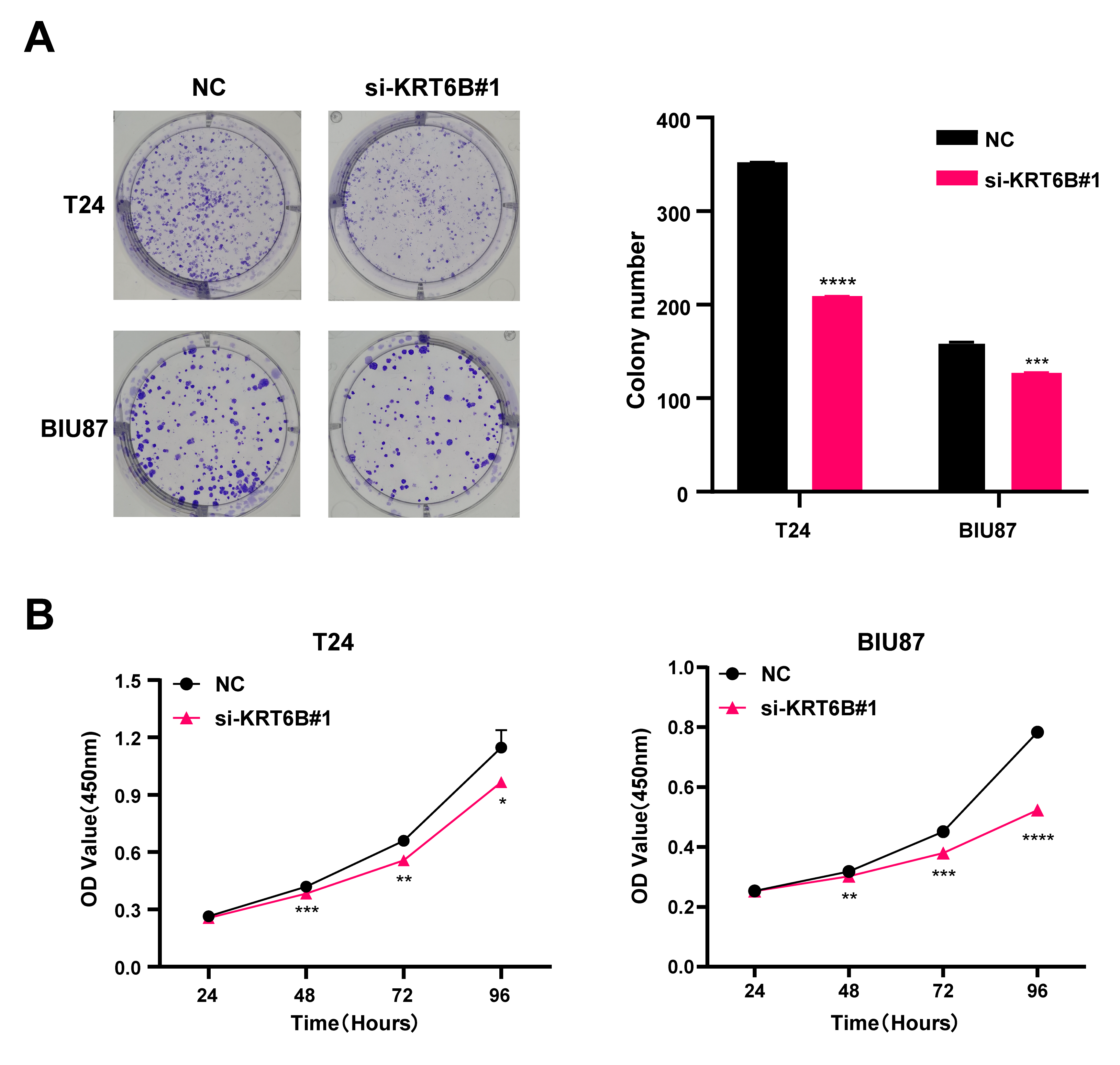

Supplement: Supplementary file 8 — Additional file 8: Figure S3. KRT6B enhanced the proliferation of BLCA cells. A Colony formation assay indicated that KRT6B could enhance the proliferation of BLCA cells. B CCK8 assay indicated that KRT6B could enhance the proliferation of BLCA cells. [file 12967_2022_3508_MOESM8_ESM.tif]
